# A bone to pick-cellular and molecular mechanisms of bone pain in sickle cell disease

**DOI:** 10.3389/fpain.2023.1302014

**Published:** 2024-01-04

**Authors:** Jahnavi Gollamudi, Kristine A. Karkoska, Oluwabukola T. Gbotosho, Wei Zou, Hyacinth I. Hyacinth, Steven L. Teitelbaum

**Affiliations:** ^1^ Division of Hematology/Oncology, Department of Internal Medicine, University of Cincinnati College of Medicine, Cincinnati, OH, United States; ^2^ Department of Neurology and Rehabilitation Medicine, University of Cincinnati College of Medicine, Cincinnati, OH, United States; ^3^ Department of Medicine, Division of Bone and Mineral Diseases, and Department of Pathology and Immunology, Division of Anatomic and Molecular Pathology, Washington University School of Medicine, St. Louis, MO, United States

**Keywords:** sickle cell disease, pain, nervous system, inflammation, bone remodeling, hemolytic anemia

## Abstract

The bone is one of the most commonly affected organs in sickle cell disease (SCD). Repeated ischemia, oxidative stress and inflammation within the bone is largely responsible for promoting bone pain. As more individuals with SCD survive into adulthood, they are likely to experience a synergistic impact of both aging and SCD on their bone health. As bone health deteriorates, bone pain will likely exacerbate. Recent mechanistic and observational studies emphasize an intricate relationship between bone remodeling and the peripheral nervous system. Under pathological conditions, abnormal bone remodeling plays a key role in the propagation of bone pain. In this review, we first summarize mechanisms and burden of select bone complications in SCD. We then discuss processes that contribute to pathological bone pain that have been described in both SCD as well as non-sickle cell animal models. We emphasize the role of bone-nervous system interactions and pitfalls when designing new therapies especially for the sickle cell population. Lastly, we also discuss future basic and translational research in addressing questions about the complex role of stress erythropoiesis and inflammation in the development of SCD bone complications, which may lead to promising therapies and reduce morbidity in this vulnerable population.

## Introduction

Sickle cell disease (SCD) is a monogenic red cell disorder that affects over 100,000 people in the United States and millions more worldwide (1, 2). A single base pair substitution at the sixth amino acid position of the beta globin gene, results in mutated hemoglobin (known as hemoglobin S, HbS) which polymerizes and changes the red cell shape (“sickle”) under hypoxic conditions. The hallmark complication of SCD is pain due to aggregation of sickled red cells within the microvasculature of the long bones, resulting in ischemia, and pain.

In addition to acute pain, repeated cycles of ischemia and infarction contribute to sickle cell bone disease (SBD). SBD refers to a combination of pathologies such as osteonecrosis, low bone mineral density, vertebral bone deformities, pathological fractures and osteomyelitis (3). SBD can contribute to chronic pain. For example, presence of osteonecrosis is associated with increased hospitalizations for vaso-occlusive episodes (VOE) requiring parenteral opiates (4).

As more individuals with SCD survive into adulthood, they are likely to experience a synergistic impact of both aging and sickle cell disease on their bone health. As bone complications accrue, the prevalence of chronic pain is also likely to increase (5). While opiates and non-steroidal anti-inflammatory drugs help with acute pain, these agents are less effective in treating chronic pain (6). Given the adverse effects of opiates including dependence (7), it is critical to understand the pathogenesis of SBD to catalyze the development of alternatives to opiates. Recently, several animal and clinical studies have elucidated contributors to the pathogenesis of chronic bone pain. While most of the studies are in non-SCD models, they may still be pertinent in SCD. In this review, we first provide a brief overview of select bone complications that result in chronic pain, and summarize mechanisms of bone pain, drawing particular attention to the role of bone remodeling, afferent nerve sensitization, and nerve sprouting. Finally, we briefly discuss tentative therapies that may have a role in bone pain in SCD.

### Low bone mineral density

One of the most common bone complications of SCD is low bone mineral density (BMD) or low bone mass (3). Low bone mass is observed in more than 50% of children (8, 9) as well as over 70% of adults with SCD (10, 11). Furthermore, in children, low BMD associates with an increased risk of avascular necrosis (AVN) and chronic pain (9). In adults with SCD, low bone mass has been strongly linked to risk of fractures at an early age (12).

Mechanistic studies show low BMD in SCD is due to both accelerated bone loss and poor bone formation. Animal studies show increased marrow osteoclast precursors numbers and activity which is responsible for bone loss (13). In addition, defective terminal osteoblast differentiation results in poor bone formation (13–16). Markers of increased osteoclast activity such as tartrate-resistant acid phosphatase (TRAP) type 5b, have also been observed in sera of individuals with SCD (17–19). Preclinical, and clinical studies show osteoclast activation associates with both pathological bone loss and chronic bone pain (20–23). Interestingly, children with SCD and low BMD had improvement in chronic pain after receiving bisphosphates, an anti-resorptive medication, highlighting the role of OC activity in both low BMD and pain (24).

### Fractures

Fractures are common *n* in children and young adults with SCD (11, 24). In one study, 46% of adults had evidence of vertebral fractures (11, 12). The mean age of adults with fractures is often less than 40 years (11, 12). Fractures can be seen in upper extremities, vertebral, pelvic, and femoral bone (11, 12, 25, 26). Risk factors for developing fractures, in addition to male sex and low vitamin D levels (27), include elevated lactate dehydrogenase (LDH) and elevated aspartate aminotransferase (AST) (12), suggesting a role for hemolysis. There was a lack of correlation with bone mineral density (11, 28). Furthermore, transfusions did not affect the risk of fractures (27). Presence of fractures and abnormal healing from fractures can worsen pre-existing pain and contribute to chronic pain (29). The current treatment for fractures is mostly conservative and surgeries such as laminectomy are reserved for when conservative management fails (30, 31).

Mechanistically, decreased bone strength results from loss of cortical, trabecular, and abnormal bone matrix composition known as bone quality (32). In two separate studies, the authors show femurs from sickle mice required less force to deform and had lower capacity to sustain high mechanical stress suggesting increased fragility and risk for fractures (14, 15). As in humans, there is a lack of correlation with BMD. This suggests that current imaging methods assessing BMD may be insufficient to inform fracture risk and capture complex changes in bone quality (33).

### Avascular necrosis

Avascular necrosis or osteonecrosis is the second most common chronic complication of SCD (4). About 10%–22% of patients with SCD are affected; however, in reality, prevalence is likely to be much higher (17, 34).

Avascular necrosis results from a disruption of the blood supply to the ends of long bones, which results in death of bone tissue (35). In SCD, femoral or humeral heads are commonly affected. The femoral head is particularly susceptible to ischemia as it lacks a collateral blood supply. Risk factors include older age, male sex, high hemoglobin, body mass index, leukopenia, frequent VOEs, and a history of acute chest syndrome (36). Furthermore, genetic polymorphisms in genes with roles in vascular integrity, inflammation, and oxidant stress, such as *Klotho*, bone-morphogenic protein 6 *(BMP-6)*, and annexin 2 *(ANX2)*, confer an increased risk of AVN in SCD (37). While initially asymptomatic, AVN can rapidly progress to collapse and narrowing of the joint space (38), particularly in SCD. Treatment for AVN includes improving vitamin D status, physical therapy and/or surgical interventions, however the optimal treatment modality for early AVN is still debated (39).

### Bone remodeling, innervation, and its functions

Bone is a dynamic tissue maintained in homeostasis by opposing actions of bone resorption and formation. The cells responsible for this homeostasis include osteoclasts, osteocytes, and osteoblasts (40). Osteoclasts arise from a myeloid lineage (32). Osteoblasts arise from mesenchymal stromal cells (MSC) and are primarily involved in bone formation at sites resorbed by osteoclasts (40). Osteocytes are terminally differentiated osteoblasts that form the bone matrix (40). The balance of bone resorption and formation is governed by cytokines, local bone environment and precise crosstalk between osteoclasts, osteoblasts, and osteocytes (41). For example, osteoblasts regulate formation, activation, and maturation of multinucleated osteoclasts from their precursors by secreting macrophage colony stimulating factor (M-CSF), receptor activator of nuclear factor-κB ligand (RANKL) and osteoprotegerin (OPG) (32). Osteocytes, which are the principal source of RANKL, send precise signals which allows for osteoclasts to resorb bone at designated locations (42). Osteoclasts can then resorb bone by secreting enzymes such as cathepsin K and hydrogen ions (41).

Bone is richly innervated (43). The skeletal nerves can be classified as sensory, sympathetic, or parasympathetic nerves (44). The density of the nerves is highest in the periosteum followed by bone marrow and lowest in mineralized bone (45). Sensory or afferent nerves are either thinly myelinated (A-delta) or unmyelinated (C-fibers) and transmit pain from bone and bone marrow to the dorsal root ganglion (DRG) (46–48). Together, A-delta and C-fibers account for most of the pain-transducing nerves in the bone (43). Specifically in SCD, skeletal pathologies such as fractures, marrow infarction, and inflammation may play a role in the initiation and propagation of bone pain.

Noxious stimuli activate pain receptors on sensory neurons such as acid-sensing ion channel 3 (ASIC3), transient receptor potential channel vanilloid subfamily member 1 (TRPV1), tetrodotoxin (TTX)-resistant sodium channels (Nav.1.8), purinergic receptor (P2X3), endothelin receptor (ETAR), prostaglandin (PG) among others (22, 49). Upon activation of these receptors, neurotransmitters such as calcitonin gene–related peptide (CGRP), substance *P* (SP), glutamate and pituitary adenylate cyclase-activating polypeptide (PACAP) are released from nerve terminals in the DRG (45, 50, 51). Skeletal afferent neurons also express tropomyosin receptor kinase A positive (TrkA+) (52). In fact, the majority of bone-innervating sensory fibers are CGRP and TrkA positive C-fiber neurons (53). CGRP and substance *P* signaling also affects bone mass (54, 55). For example, mice lacking CGRP have low bone mass, due to loss of CGRP mediated effects on osteoblasts (54, 56). Bone resorption and formation are also dependent on both sympathetic and parasympathetic signaling (44, 57). Osteoclasts and osteoblasts express α- and β-adrenergic receptors which are activated when bound to norepinephrine (NE) (58) which favors bone resorption in mice and humans (58, 59). As is the case with sensory nerves, neurotransmitters from sympathetic nerves can also modulate bone generation. Neuropeptide Y via its action on hypothalamic Y2 receptors has been shown to exert a negative effect on osteoblast activity and bone formation (60, 61). Of note, osteoblasts and to some extent osteoclasts, also secrete endocannabinoids such as 2-arachidonylglycerol (2-AG), which can reduce sympathetic signaling via CB1 receptors on skeletal sympathetic nerve endings (62). Lastly, neuropeptides from sensory nerves such as CGRP can also affect sympathetic signaling (63). Thus, there is an intricate and complex relationship between bone cells, the sensory and autonomic nervous systems. This has implications for therapies to treat bone pain, as complete inhibition of neuro-receptors may have unintended consequences on bone formation or resorption.

**Figure 1:**
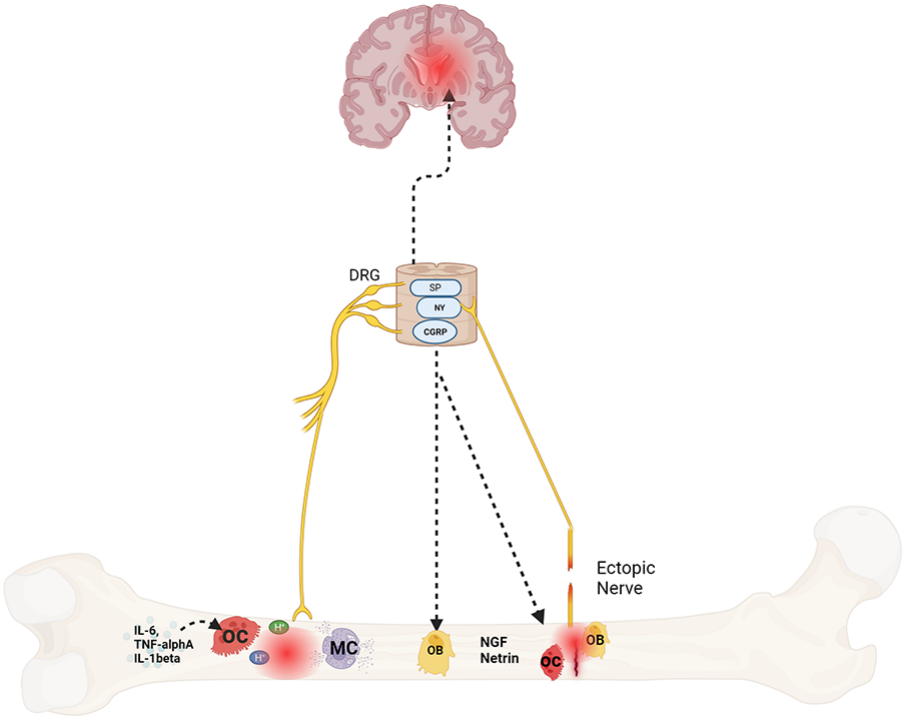
Mechanisms of bone pain in sickle cell disease. Protons secreted by osteoclasts in response to cytokines, fractures, or ischemia triggers afferent nerve signaling via nociceptors like acid-sensing ion channel 3 (ASIC3) and the transient receptor potential channel vanilloid subfamily member 1 (TRPV1) which communicate to the brain and spinal cord via neurotransmitters such as substance *P* (SP), neuropeptide Y (NY) and calcitonin gene–related peptide (CGRP). These neurotransmitters also sensitize the afferent nerves. In addition, they also play a role in bone formation and resorption as well. Lastly, mast cells (MC), osteoclasts and osteoblasts also release nerve growth factor (NGF) and netrin respectively that promotes ectopic nerve growth which further contributes to pain. Created with BioRender.com.

Emerging data suggests bone pain is due to pathologic remodeling, exuberant neurotransmitter release, and ectopic nerve growth. These abnormal changes occur with age, stress and/or systemic neuroinflammation, all of which are at play in SCD (22, 64, 65) (Figure 1). In the following sections, we provide evidence for these processes in bone pain described in SCD and non SCD models.

### Bone remodeling and bone pain

In SCD, chronic and acute inflammation results in elevated levels of circulating interleukin 6 (IL-6), interleukin 1 beta (IL-1β), and tumor necrosis factor alpha (TNF-α) (66). These cytokines promote osteoclastogenesis (67–69). Tumor necrosis factor alpha, for example, can directly increase osteoclast activity with minimal RANKL secretion (67–69). The increased osteoclastic activity favors accelerated bone resorption. Osteoclasts resorb bone by acidifying their immediate bone environment (41). In addition to mobilizing minerals, low pH (acid) is a noxious stimulus, which is sensed by nociceptors such as ASICs and 1TRPV1 (51, 70). In preclinical models, a reduction in osteoclast activity was correlated with a decrease in ASICs and TRPV1 expression, activation of neurons in DRG and decreased pain behaviors (21). Furthermore, in this study and others, inhibition of ASIC3 and TRPV1 resulted in decreased pain and biomarkers for early osteoblast differentiation (71, 72). This is an important point as prolonged inhibition may potentially result in decreased bone formation and delayed fracture healing (72). Lastly, complete abrogation of these receptors, may have unintended side effects. For example, in an animal model of SCD, complete abrogation of TRPV1 resulted in worsening of VOEs by attenuating vasodilatory effects of CGRP (73).

In addition to inhibiting nociceptors, limiting osteoclast activity also reduces bone pain. Numerous studies in cancer and osteoporosis models show that bisphosphonates, which reduce osteoclast activity, improve bone pain (74–76). Pre-clinical studies in mouse models of SCD also show that bisphosphonates reduce markers of bone turnover and improve bone microarchitecture (13). A recent study in 46 children with SCD-related skeletal morbidity showed improvement in chronic pain after treatment with intravenous bisphosphates, highlighting the possible interaction between osteoclast activation, abnormal bone remodeling, and the development of bone pain (24). An ongoing prospective study is evaluating the effect of bisphosphonates on bone pain in adults with SCD (NCT05283148).

### Afferent nerve sensitization and bone pain

A vast majority of the skeletal nerves express TrKA, whose ligand is nerve growth factor (NGF) (52). NGF promotes proliferation and survival of sympathetic and sensory afferent neurons during development (77). TrKA/NGF signaling also plays a critical role in bone fracture repair (78). However, abnormal TrkA/NGF signaling in the setting of exuberant inflammation results in peripheral nerve sensitization. Experimental evidence has shown that NGF is also released by mast cells (79, 80), lymphocytes (81), and monocytes/macrophages (82) in response to tissue inflammation. NGF propagates peripheral nerve sensitization through several mechanisms: (1) NGF binding to TrkA + neurons increases neuronal excitability by altering the activity of different ion channels in the nerve ending (83), which results in a lower threshold for neuronal depolarization. (2) NGF/TrKA complexes promote gene expression of nociceptors such as TRPV1, ASIC, Nav1.8 (sodium) channels, CaV (calcium channels), in addition to release of neuropeptides such as CGRP, and substance *P*, which can, in turn, increase the sensitivity of the afferent neuron to NGF (84). and (3) NGF induces inflammatory cells to release bradykinin, histamine, ATP, serotonin, and protons, which in turn activate receptors and ion channels thus propagating the pain feedback loop (85).

In rodent models of osteoarthritis or fracture repair, an increase in NGF levels associated with increased pain behaviors (86, 87). Furthermore, local, and systemic NGF administration into healthy humans induces deep pain and hypersensitivity that can last for several days (88, 89). Also, individuals with SCD and chronic pain also show elevated circulating levels of NGF (90). Thus, taken together, therapies that affect inflammatory cell release of NGF or inhibit NGF may be beneficial in decreasing bone pain.

Evidence from SCD and non-SCD models (91, 92) corroborate inhibition of NGF as a target to reduce pain. A murine model of SCD underscored the importance of mast cell activation, infiltration and NGF in pain hypersensitivity by showing that tryptase, secreted by mast cells, promotes release of substance *P* and CGRP in DRG (targets of NGF), and inhibition of mast cell activation by imatinib resulted in reduced musculoskeletal pain behaviors and reduced activation of neurons in the DRG (92). Animal models of osteoarthritis also show that increased mast cell activation and numbers resulted in worsening inflammation and pain which can be reversed with mast cell inhibition. Thus, mast cell inhibition may help with bone pain (93) and is a potential therapeutic target. Therapies such as monoclonal antibodies against NGF (94) showed improvement in OA induced pain in preclinical and clinical trials (95). However, clinical trials were halted early due to reports of rapidly progressive OA and of osteonecrosis among individuals receiving these agents. Preclinical studies suggest it could be due to autonomic dysfunction or decreased angiogenesis (52, 96) however, some studies speculate that it could be the natural progression of the disease itself (91).

### Nerve growth sprouting and bone pain

Bone innervation and bone remodeling are closely linked (44). Physiologically, nerve growth is an important signal for ossification of newly formed bone (44). However, under pathological states, excessive nerve sprouting can result in pain hypersensitivity. Preclinical models of fractures or low back pain show that osteoblasts and osteoclasts secrete NGF and Netrin respectively, which are key proteins propagating nerve growth (78, 97, 98). In mammals, Netrins are either secreted (Netrin-1, -3, -4, and -5) or anchored to cell membrane (Netrin-G1 and -G2) (99–101). Netrin-1 is responsible for neuronal migration as well as axonal growth (102, 103). In addition to guiding neurons, Netrin-1 can also guide leukocytes to the site of injury and promote macrophage differentiation (103, 104). It is plausible netrin levels may be affected in SCD. To date, no study has surveyed the levels as well as impact of Netrin on chronic pain in preclinical models or patients with SCD.

In non-SCD model of low back pain, osteoclasts secrete Netrin-1 which supports the ingrowth of CGRP positive nerve fibers at the endplates of vertebral bodies. Elimination of osteoclasts attenuated pain behaviors (97). In an osteoarthritis model, osteoclasts were also shown to secrete Netrin-1 that induces growth of CGRP positive nerves and DRG activation (98). More importantly, both the genetic and pharmacological blockade of Netrin1 reduce pain behaviors (98) suggesting an important role for Netrin-1 inhibitors in bone pain. It is interesting to note that therapies affecting global netrin inhibition may affect other cell lines as netrin is important for hematopoietic stem cell (HSC) quiescence, and self-renewal (105). This is important as SCD is characterized by exuberant increase in HSC cells due to chronic stress erythropoiesis and inflammation resulting in premature aging of the HSC system (106).

### Future directions

Bone is a dynamic organ. Bone formation and absorption are influenced by local as well as systemic factors including inflammation (107). In SCD, chronic hemolysis, inflammation, delayed puberty, and poor muscle mass may contribute to excessive bone remodeling and loss (3). Thus, an understanding of mechanisms leading to bone loss is important to understand bone pain.

Questions that still need answering include how stress erythropoiesis contributes to bone remodeling and pain. Individuals with thalassemia, a red cell disorder characterized by defective beta or alpha globin production and ineffective erythropoiesis, also exhibit low bone mass, increased risk of fractures, and bone pain (108). Erythropoiesis can also stimulate myelopoiesis, which in the right bone microenvironment, differentiate to osteoclasts (109). Marrow expansion can also activate mechanosensitive PIEZO receptors which may transduce pain (47). Other questions include if chronic hemolysis also contributes to osteoclastogenesis. In SCD, hemolysis results in release of free heme, which contributes to reactive oxidative stress (ROS) (110). In response to ROS, cells lining the blood vessels express heme oxygenase (HO-1) which attenuates inflammation. Non-SCD models show HO-1 regulates osteoclastogenesis by inhibiting RANKL- induced osteoclastic differentiation (111). Ferroptosis, iron induced cell death, could also play a role in osteoclastogenesis, however further research is needed (112, 113).

Effective therapies against bone pain also have to balance their targets precisely, such that bone health is not impaired. For example, CGRP inhibitors, used effectively against migraines, may help with bone pain, however it could mediate increased vaso-occlusive crisis seen in animal models of SCD (73, 114). Thus, there is an unmet need for therapies that treat bone pain that are safe and do not further impact bone health in SCD.

## Conclusion

Bone complications and bone pain often starts early in childhood (4, 9) and results in much disability, missed school or work days and poor quality of life due to lack of effective treatments. The etiology is poorly understood and is likely multifactorial with contributions from hypoxia, inflammation, nutritional status, and growth delays. Interestingly, disease modifying therapies such as chronic red cell exchanges and even hematopoietic stem-cell transplant does not seem to reverse chronic pain suggesting a role for peripheral and/or central nerve sensitization (64, 115). While opiates are often used for this indication, their adverse effects limit their use.

To identify novel therapeutics, a deeper understanding of pathogenesis of bone pain is needed. Specifically, the role of systemic inflammation, central sensitization, growth delays, nutritional interventions and physical activity are lacking. Such research, for example, could catalyze the use of existing anti-inflammatory treatments often used to treat bone pain in inflammatory arthritis (116). While newer studies are investigating the role of bisphosphonates on bone pain, given complex pathogenesis of bone pain, a monotherapy may not be sufficient. Instead, an integrated and interdisciplinary approach that involves patient education, hematologists, pain specialists, nutritionists, and psychologists may be needed to help with bone pain.
